# Trends in Early and Late Midlife Substance Use through 2024 from the US Monitoring the Future Panel Study

**DOI:** 10.1093/geroni/igaf122.4037

**Published:** 2025-12-31

**Authors:** Megan Patrick, Yvonne Terry-McElrath, Joy Bohyun Jang

**Affiliations:** University of Michigan, Ann Arbor, Michigan, United States; University of Michigan, Ann Arbor, Michigan, United States; University of Michigan, Ann Arbor, Michigan, United States

## Abstract

Longitudinal data collected across the life course provide unique opportunities to understand how risk factors for health in older adulthood evolve over time. The Monitoring the Future (MTF) Panel study surveys national US samples of ∼20,000 adults each year, including a new nationally-representative cohort of 12th grade students every year since 1976 (Patrick et al., 2025). The current study reports on changes over time in substance use among those in early midlife (ages 35-50) and late midlife (ages 55-65) through 2024, focusing on cannabis and nicotine use. Cannabis use overall remains at/near all-time high levels in these age groups, as prevalence levels have doubled or nearly doubled over the past 5 and 10 years (e.g., early midlife past 12-month use increased from 4.4% in 2014 to 26.6% in 2024). The prevalence of vaping cannabis in the past 12 months reached historic highs in both age groups, rising from 8.7% in 2023 to 10.9% in 2024 among early midlife adults and from 3.5% to 5.1% among late midlife adults. Regarding nicotine, the prevalence of nicotine pouch use increased from 3.3% in 2023 to 4.9% in 2024 in early midlife, and from 2.2% to 4.3% for late midlife. Among late midlife adults there were significant increases in the use of hookah (from 2.0% to 3.4%) and smokeless tobacco (from 3.3% to 5.7%) in the past 12 months from 2023 to 2024. These shifts illustrate how substance use is evolving among adults, with implications for health and wellbeing in midlife and beyond.

